# Organic π-type thermoelectric module supported by photolithographic mold: a working hypothesis of sticky thermoelectric materials

**DOI:** 10.1080/14686996.2018.1487239

**Published:** 2018-07-17

**Authors:** Norifusa Satoh, Masaji Otsuka, Tomoko Ohki, Akihiko Ohi, Yasuaki Sakurai, Yukihiko Yamashita, Takao Mori

**Affiliations:** a Center for Functional Sensor & Actuator, National Institute for Materials Science (NIMS), Tsukuba, Japan; b Research Network and Facility Services Division, National Institute for Materials Science (NIMS), Tsukuba, Japan; c Denka Innovation Center, Denka Company Limited, Tokyo, Japan

**Keywords:** Contact resistance, flexible thermoelectric sheet, mass-production, inverse materials design, 50 Energy Materials; 210 Thermoelectronics / Thermal transport / insulators

## Abstract

To examine the potential of organic thermoelectrics (TEs) for energy harvesting, we fabricated an organic TE module to achieve 250 mV in the open-circuit voltage which is sufficient to drive a commercially available booster circuit designed for energy harvesting usage. We chose the π-type module structure to maintain the temperature differences in organic TE legs, and then optimized the p- and n-type TE materials’ properties. After injecting the p- and n-type TE materials into photolithographic mold, we eventually achieved 250 mV in the open-circuit voltage by a method to form the upper electrodes. However, we faced a difficulty to reduce the contact resistance in this material system. We conclude that TE materials must be inversely designed from the viewpoints of the expected module structures and mass-production processes, especially for the purpose of energy harvesting.

## GRAPHICAL ABSTRACT

**Figure:**
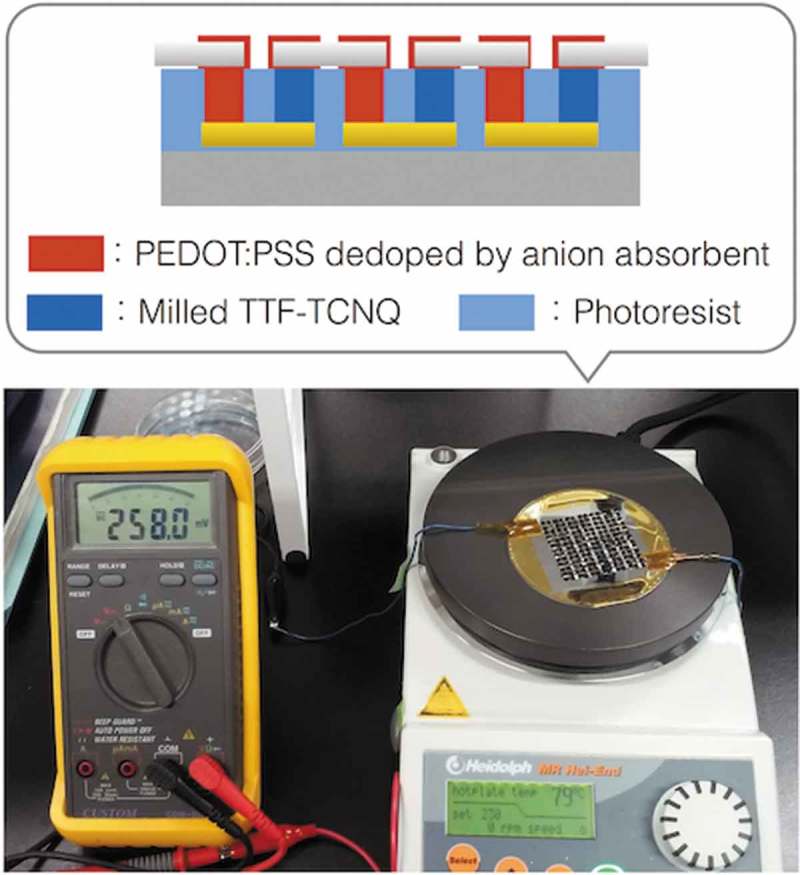

## 1. Introduction

Thermoelectrics (TEs) have the potential to recover usable energy from waste heat and capture convenient energy from the environment [1–3]. The largest amount of heat is wasted in the low temperature region, ~ 150 °C [4], because the conventional systems do not have recovery capacity for such low temperature heat. In contrast, TEs are expected to exceed the conventional systems in the energy convergent efficiency at the low temperature region [5]. In addition to the technical advantage, organic materials can provide an additional value for TEs: flexibility. To absorb thermal energy from heat elements, TE modules need to confirm their shape to that of heat elements. In the case of solid inorganic TE modules, therefore, we have to fabricate the TE modules one by one to confirm their shapes to the individual heat elements even though heat elements have large variety of the shapes in our environment. It has been one of the biggest issues inhibiting TEs from a wide usage for energy harvesting. Considering the amount of waste heat and the demand for energy harvesting, we must mass-produce TE modules at reasonable cost [6]. In contrast, flexibility of organic TE modules allows the economic mass-production, for example, using roll-to-roll processes, because the flexible TE modules can adjust their shapes to fit various heat elements even after being mass-produced. Although the difficulty in the measurement of in-plain thermal conductivity of organic films may raise some question of the accuracy [7], the TE figure of merit (*ZT*) of organic materials has been reported to reach up to 0.42, recently [2,8,9]. *ZT* is given as:

(1) $$ZT=\frac{S^{2}\sigma }{\kappa }T$$

where *S, σ, κ*, and *T* are Seebeck coefficient, electric conductivity, thermal conductivity, and absolute temperature, respectively. These TE properties have tradeoffs, and many efforts are being made to improve them [10,11]. Flexible inorganic-organic hybrid materials are also proposed to achieve high *ZT* approaching to 0.3 [12–14]. The *S*, yet, generates only several 10 µV/K for typical organic materials. To drive electric devices with booster circuits, we need to pattern and connect more than 100 TE legs at low cost as a TE module, using mass-producible processes.

**Figure 1.**
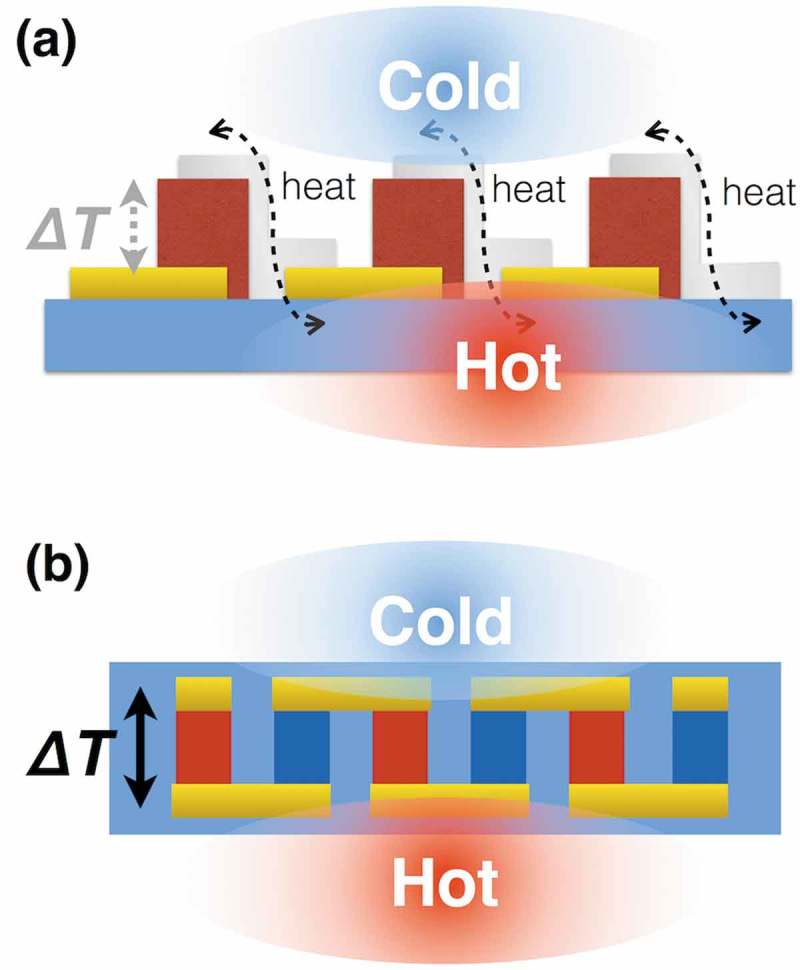
Comparison of TE module structures to absorb heat energy from large area: (a) single-leg TE module structure; (b) π-type TE module structure. Flexible TE sheets necessitate low-*κ* TE materials like organics and the π-type TE module structure to create Δ*T* in the sheet thickness.

**Figure 2.**
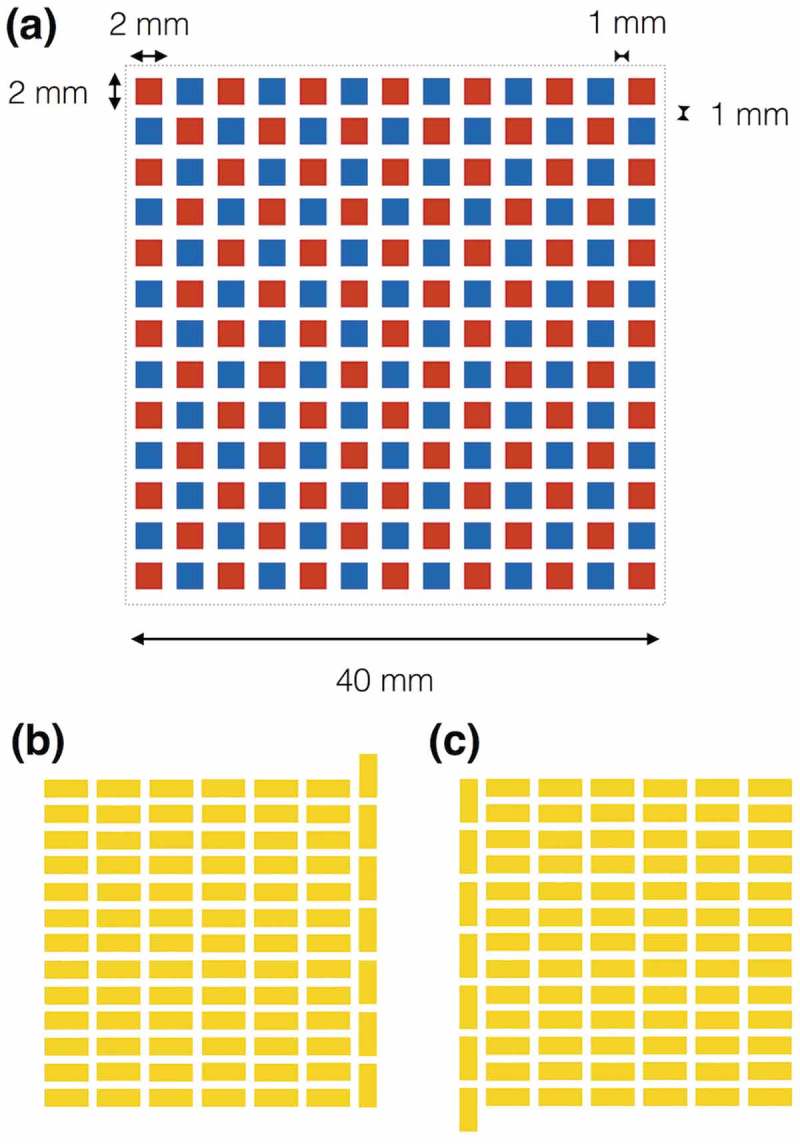
The module pattern of 13 × 13 legs in 40 × 40 mm^2^ aiming to drive electric devices with a booster circuit: (a) p-type TE leg (red) and n-type TE leg (blue) pattern; (b) bottom electrode pattern; (c) upper electrode pattern. The designed module is expected to drive the booster circuit when the output voltage of single π-unit reaches 3 mV.

To examine the potential of organic TE modules fabricated only using the common processes applied in mass-productions, herein, we utilize a photolithographic mold to pattern organic TE legs. As a simple way, we can fabricate organic one-leg TE modules via printing the bottom electrodes, the organic TE materials, and the upper electrodes connecting to the adjacent bottom electrodes (Figure 1(a)). However, the organic single-leg TE modules cannot generate electricity efficiently because the upper electrodes conduct the heat from the adjacent bottom electrodes and decrease the temperature difference (*ΔT*) for the TE generation. To maintain the *ΔT* by separating the upper and bottom electrodes, we fabricate π-type TE module structure via fulfilling p-type and n-type TE organic materials into the photolithographic mold (Figure 1(b)). Aiming to drive electric devices with a booster circuit, we design a module pattern of 13 × 13 legs in 40 × 40 mm^2^ (Figure 2).

In this study, we first optimize p-type and n-type TE materials based on the common organic materials, poly(3,4-ethylenedioxy thiophene) polystyrene sulfonate (PEDOT:PSS) and tetrathiafulvalene 7,7,8,8-tetracyanoquinodimethane salt (TTF-TCNQ) [15,16]. Although dedoping of PSS yielded *ZT* = 0.42 in PEDOT:PSS [9], the demonstrated process cannot be implemented as an economic mass-production process because the spin-cast PEDOT:PSS must be immersed once in ethylene glycol for 1 h for every 20–125 nm deposition; the organic TE legs need at least 20 µm thickness to maintain the internal Δ*T*. Therefore, we also examine dedoping of PSS using an anion absorbent, where the anionic sulfonic group of PSS should be captured in a mixture solution with the anion absorbent before being injected into the photolithographic mold. Related to the material optimization, we also report detailed preparatory conditions of the n-type TE leg material because the previous reports only describe the mixing ratio of TTF-TCNQ and polyvinylchloride (PVC) [15,16]. When a single π-unit reaches 3 mV, the designed module can reach 250 mV to drive electric devices with a commercially available booster circuit designed for energy harvesting usage. The current Bi_2_Te_3_-based TE modules also need such booster circuits to drive electric devices, especially in the low-temperature region for the energy harvesting purpose.

**Figure 3.**
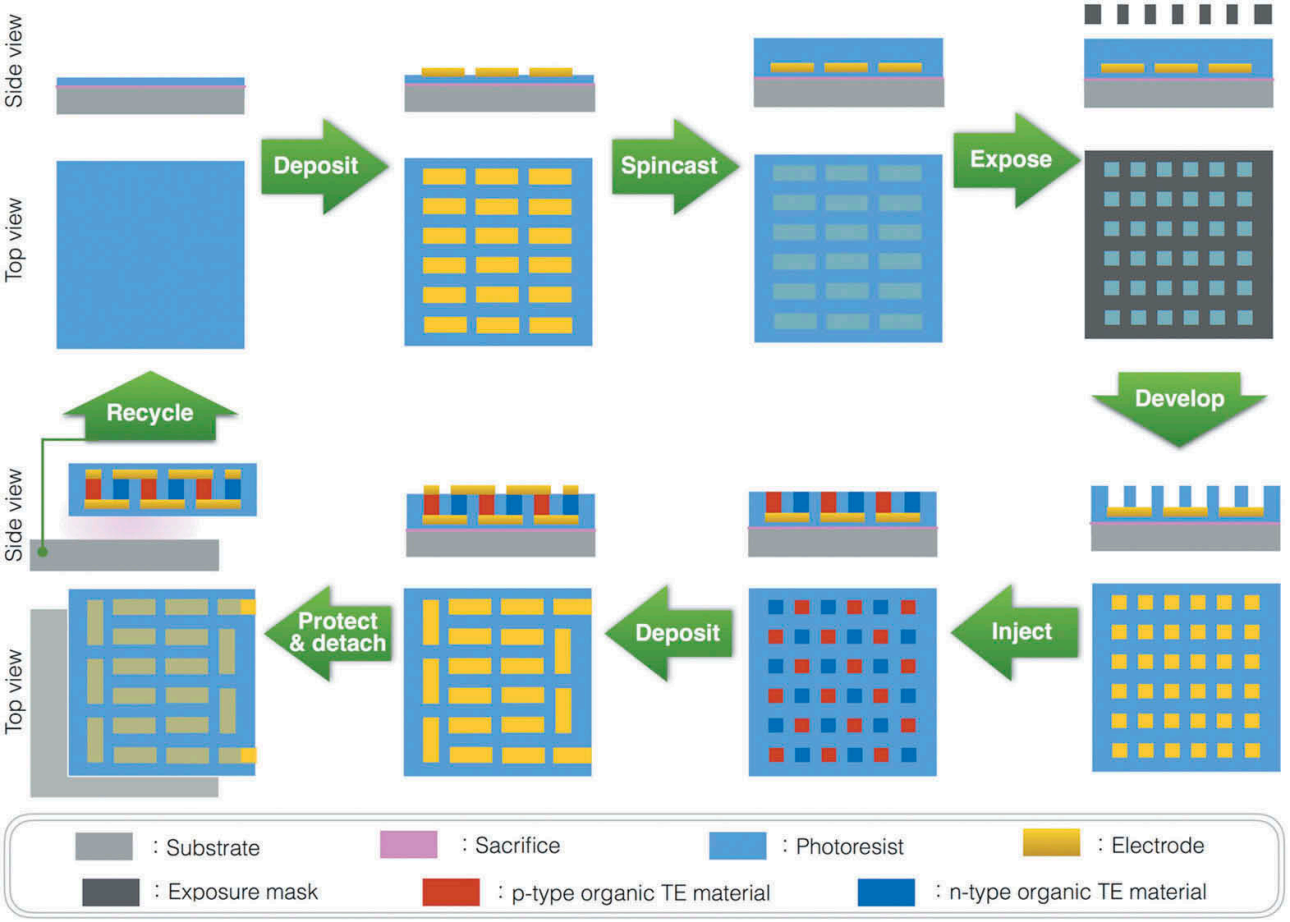
Schematic fabrication process of the organic π-type TE module based on well-established techniques.

To finalize the π-type TE module structure, we secondly adopt two methods to form the electric contacts on the upper side of module. The first method is depositing Au electrodes from the vapor in a vacuum and the other method is pasting electrically conductive adhesives (ECAs), such as PEDOT:PSS and Ag paste. As a foundation, good electric contact necessitates two conditions; (1) both electrodes and TE materials having high *σ* and (2) they physically contacting each other well at the interface. During the vacuum deposition, in principle, atomic Au vapor reaches the surface of the TE materials and condenses into small droplets to build up the electrodes from the atoms. Thus, we can expect a good electrical contact as confirmed in a molecular electronics example of Au/PEDOT:PSS/Au [17]. According to the reported results, the contact resistance should be less than 0.02 Ω for this module design. In a slightly different way, we can also expect that ECAs support good electric contacts. Since the surface of materials is not atomically flat except for rare cases, we cannot create good electric contacts, for example, on the TE materials in an economic manner. The ECAs can fill the interfacial gaps caused by the rough surfaces of electrodes and TE materials, improving the electric contacts. Overall, we attempt to re-arrange well-established fabrication processes, such as photolithography, fulfilling, and electrode deposition, to emergently fabricate the organic π-type TE modules (Figure 3).

## 2. Experimental section

### 2.1. Optimizing the organic TE legs

Since economic mass production is the ultimate goal, unless stated otherwise, all chemicals were obtained from commercial sources and used without further purification. The n-type TE material was prepared by milling 60 mg TTF-TCNQ in 1 ml toluene with 200 of 1-mm stainless balls and 20 of 4-mm stainless balls for 2 days, encapsulated in 5 ml vials. The particle size was measured by scanning electron microscopy (Hitachi FE-SEM S-5000, Hitachi high-technologies corporation, Tokyo, Japan). After extraction with toluene from the original PVC (high molecular weight, Aldrich, St. Louis, USA), toluene-soluble weighed PVC was added to the vials. Using the single π-unit structure with as-received PEDOT:PSS on the photolithographic mold, the output voltage of n-type TE legs was also measured with a digital multimeter (CDM-2000D, CUSTOM, Tokyo, Japan), where the Si wafer side is heated on a hot plate and the other side is naturally cooled by air.

The p-type TE material PEDOT:PSS (1% water solution, high-conductivity grade, Aldrich, St. Louis, USA) was dedoped by stirring with an anion absorbent KW-1000S (Kyowa Chemical Industry Co. Ltd., Kagawa, Japan) overnight. To measure the power factor (*PF *= *S* ^2^ *σ*), the mixture solution was cast on glass plates after the addition of 0 µl, 10 µl, 20 µl, and 30 µl dimethyl sulfoxide (DMSO) per 1 ml PEDOT:PSS solution. After being baked at 80 ^º^C in a vacuum chamber overnight, the *S* and *σ* of the films (typically 10 µm in thickness) were measured with the ZEM-3 measurement system (ULVAC Technologies Inc., Knagawa, Japan) according to the Japanese industrial standards JIS R 1650–1 and 1650–2 with a temperature variation of 0.3 K. Using the single π-unit structure with the ball-milled TTF-TCNQ on the photolithographic mold, the output voltage of p-type TE legs was measured with a digital multimeter, where the Si wafer side is heated on a hot plate and the other side is naturally cooled by air.

### 2.2. Fabrication process of the photolithographic mold and the upper electrodes

Before fabricating the TE module, ProTEK B3 (Brewer Science, Missouri, USA) was spin-cast on 3-inch Si wafer as a scarifying layer soluble to organic solvents like acetone so that the final TE module can be detached from the Si wafer. After depositing 10-nm Cr/100-nm Al layer to protect the scarifying layer from the next spin-cast of photoresist and forming 5 µm fully-exposed photoresist from diluted SU-8 3025 (Nippon Kayaku Co. Ltd., Tokyo, Japan) as an insulating layer, the bottom 10-nm Ti/100-nm Au electrodes were deposited through a stencil mask with an electron beam physical vapor deposition system (ULVAC Co. Inc. Kanagawa, Japan). As the photolithographic mold layer, ca. 70-µm photoresist of SU-8 3050 (Nippon Kayaku Co. Ltd., Tokyo, Japan) was partially exposed with a film mask to make holes for TE legs on the bottom Ti/Au electrodes. After the TE materials were injected into the mold, the Si wafer was baked at 80 ºC in a vacuum chamber overnight. The upper electrodes were formed by two methods. In the case of vacuum deposition, 100-nm Au electrodes were deposited in the same manner with the bottom electrodes except the stencil mask was rotated 180º from the original position. In the case of adhesion, Al foils were adhered with ECAs, such as PEDOT:PSS and Ag paste, to finalize the π-type TE module structure. The output voltage of the π-type TE module structure was measured with a digital multimeter before the detachment, where the Si wafer side is heated on a hot plate and the other side is naturally cooled by air.

## 3. Results and discussion

### 3.1. Optimizing the organic TE legs

**Figure 4.**
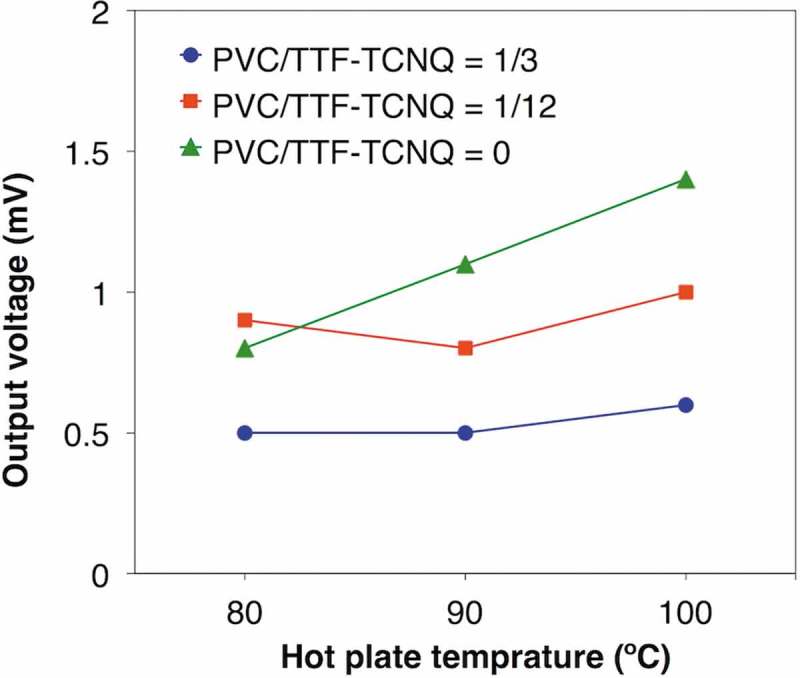
Output voltage of the single π-unit of as-received PEDOT:PSS and the ball-milled TTF-TCNQ mixed with PVC at different ratios.

We first milled the n-type TE material, TTF-TCNQ, in toluene to increase the amount injected into the photolithographic mold because TTF-TCNQ is unsolvable to common solvents. After the ball milling, the particle size decreased from ca. 100 µm to less than 1 µm, and the viscosity of n-type TE paste increased. The result indicates that smaller TTF-TCNQ particles interact with each other more strongly, which is reasonable because smaller particles have higher surface energy. Thus, we can handle the ball-milled TTF-TCNQ as an n-type TE paste without any binder in this study. Conversely, the viscosity deceased after the addition of PVC, suggesting that PVC inhibits the interaction between TTF-TCNQ particles. Using the single π-unit of the ball-milled TTF-TCNQ at the different mixing ratio of PVC with as-received PEDOT:PSS on the photolithographic mold, we found that PVC degrades the TE performances (Figure 4; see the details in Table S1). Considering that PVC decreased the paste viscosity, we surmise that PVC passivates the surface of TTF-TCNQ particles, thereby inhibiting their aggregation and worsening the TE performance.

**Figure 5.**
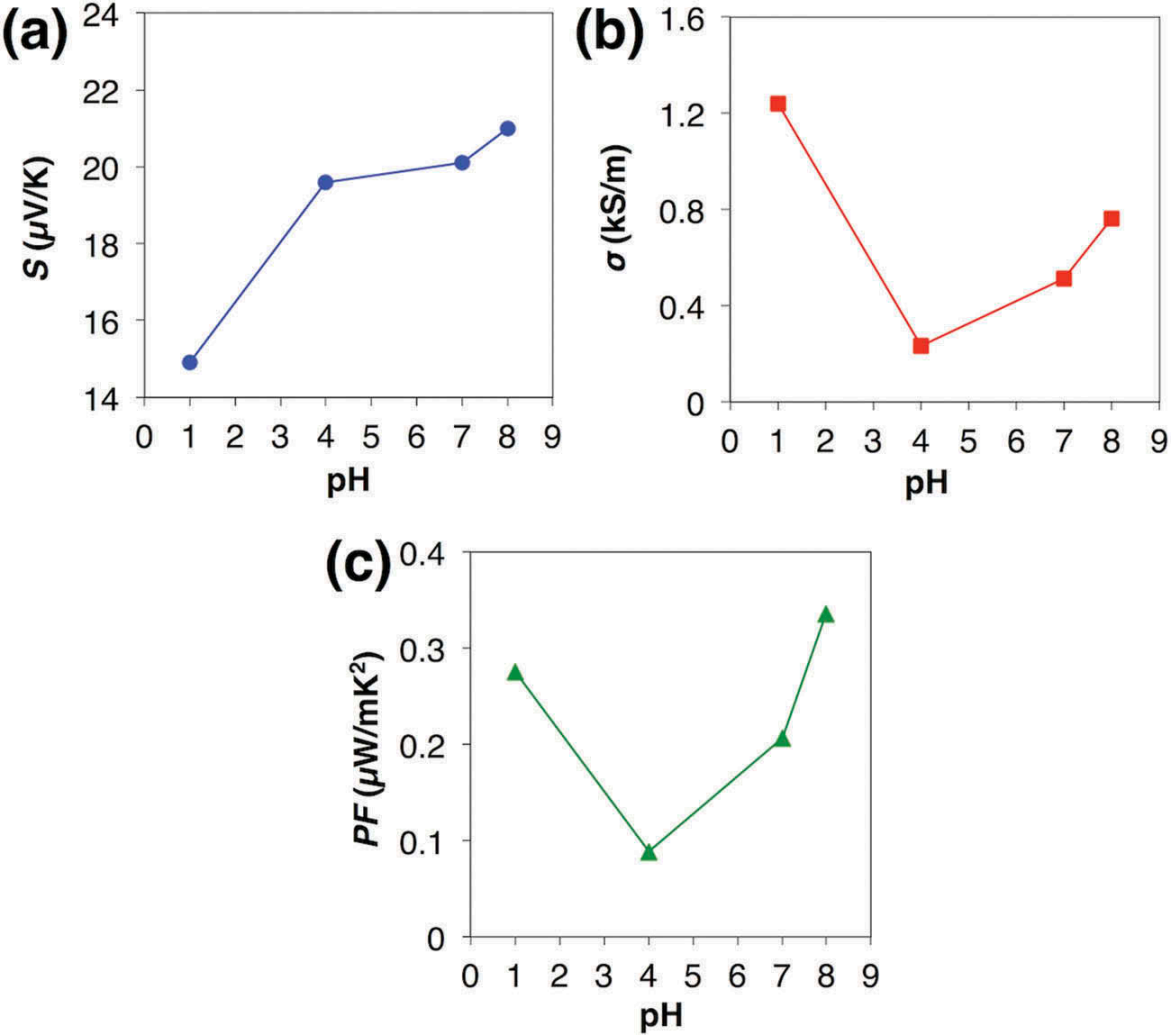
TE performances of PEDOT:PSS dedoped by KW-1000S: (a) Seebeck coefficients; (b) electric conductivities; (c) power factors.

**Figure 6.**
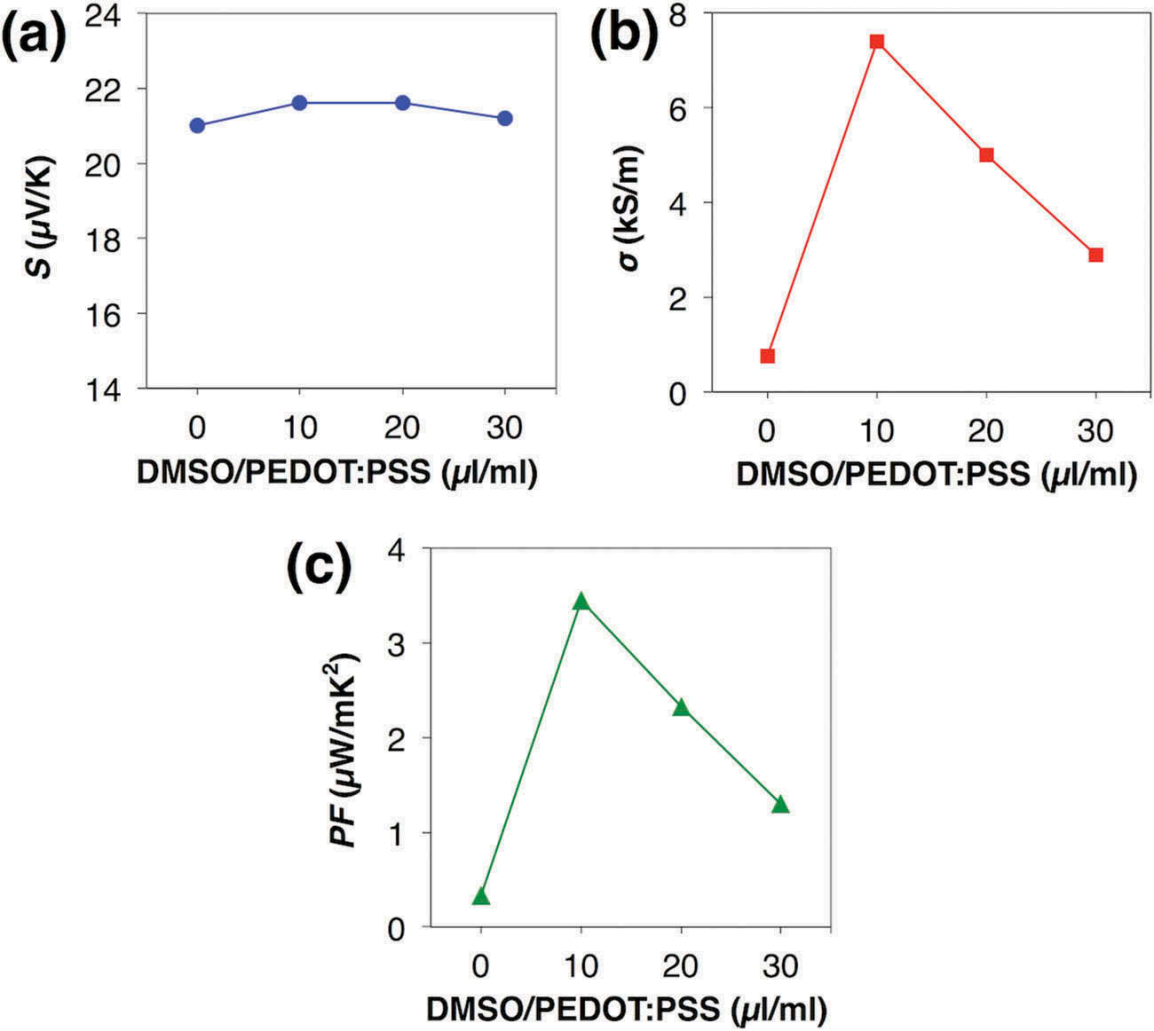
TE performances of the dedoped PEDOT:PSS after the addition of different volume of DMSO per 1 ml PEDOT:PSS solution: (a) Seebeck coefficients; (b) electric conductivities; (c) power factors.

**Figure 7.**
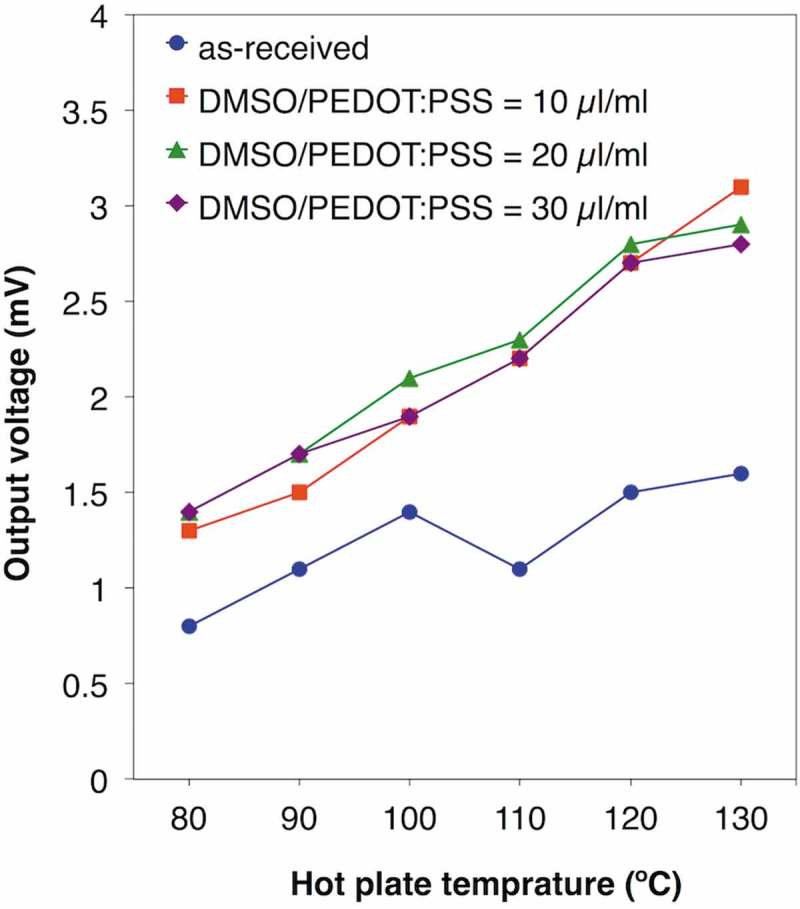
Output voltage of the single π-unit of the ball-milled TTF-TCNQ and as-received PEDOT:PSS or the dedoped PEDOT:PSS after the addition of different volumes of DMSO per 1 ml PEDOT:PSS solution.

To achieve 3 mV on the single π-unit, we chose the ball-milled TTF-TCNQ without the addition of PVC as the n-type TE material and next attempted to enhance the TE voltage via dedoping PSS from PEDOT:PSS by the anion absorbent, KW-1000S. Adding 1.75 mg, 2.38 mg, and 2.68 mg of KW-1000S per 1 ml PEDOT:PSS solution, the original pH 1 shifted to pH 4, 7, and 8, respectively. The *PF* measurement of drop-cast films also supported the idea of dedoped PEDOT:PSS (Figure 5; see the details in Table S2). As is the tendency of conductive polymers, the dedoped PEDOT:PSS first enhanced the *S* and decreased the *σ* due to the decrease in the carrier concentration. Similar to the immersed case [9], however, both the *S* and the *σ* were enhanced in the region from pH 4 to pH 8, which can be explained by the removed PSS shortening electron-tunneling distance between the conductive PEDOT chains and enhancing the carrier mobility. In total, the dedoped PEDOT:PSS improved the *PF* from 0.275 µW/mK^2^ at pH 1 to 0.336 µW/mK^2^ at pH 8. To recover the *σ*, we further added DMSO to the dedoped PEDOT:PSS, where DMSO was expected to align the PEDOT conductive chains [9]. Although it did not affect the *S*, DMSO improved the *σ* and reached 3.45 µW/mK^2^ in the *PF* (Figure 6; see the details in Table S3). As a result, we were able to achieve 3 mV on the single π-unit using the single π-unit of the ball-milled TTF-TCNQ and the DMSO-mixed dedoped PEDOT:PSS (Figure 7; see the details in Table S4). Thus, we predict the 13 × 13 legs module reaches 250 mV, a criterion to drive a booster circuit.

### 3.2. Upper electrodes finalizing the organic π-type TE module

To finalize the organic π-type TE module, we first attempted to form Au electrodes by electron beam physical vapor deposition because we can estimate low contact resistance less than 0.02 Ω for this module design based on the experimental data reported in the literature [17]. However, we realized that the p-type TE legs of the DMSO-mixed dedoped PEDOT:PSS were peeled off in the vacuum chamber of deposition system. Since the dedoped PEDOT:PSS without DMSO did not peel off, we assume that remaining DMSO in PEDOT:PSS vaporizes from the surface in the vacuum chamber, then shrinks the surface, and finally causes the mechanical tension to peel off. Thus, we further scanned the prebake condition but could not stop the peeling off. To deposit the upper Au electrodes, in this study, we temporally used the dedoped PEDOT:PSS without DMSO. After depositing the upper Au electrodes, we confirmed the electric connection through the 13 × 13 legs. However, the organic π-type TE module only showed 0.8 mV at 80 ºC even though accumulating the voltage through the 13 × 13 legs.

**Figure 8.**
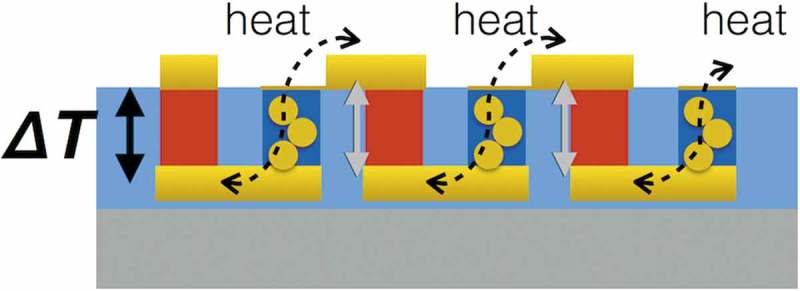
Schematic of the Au penetrated π-type TE module. The Au decreases *ΔT* of the penetrated n-type leg itself and the next p-type leg due to the heat conduction. Therefore, only the first p-type leg generates electricity.

The non-accumulating voltage is caused by Au vapor penetrating into the ball-milled TTF-TCNQ; we confirmed that the gold color covering the TTF-TCNQ particles reached the bottom Au electrodes (Figure 8). Similar to the organic single-leg TE modules, the penetrated Au conducts heat from the bottom electrodes to the upper electrodes and decreases *ΔT* for all the TE generations except for the first p-type leg’s one. If PVC filled the space between the ball-milled TTF-TCNQ particles, Au vapor might not penetrate the n-type TE legs. In any case, however, the TE voltage does not reach 250 mV in this material system. Overall, we had expected Au vapor deposition to give low contact resistance but it turns out to be a poor match with the important factors for the legs’ performance optimizations, such as having DMSO addition to PEDOT:PSS and the ball-milled TTF-TCNQ without PVC. This result suggests that we need to explore non-volatile additives to PEDOT:PSS [18] and polymer-based n-type materials soluble to common solvents [19].

**Figure 9.**
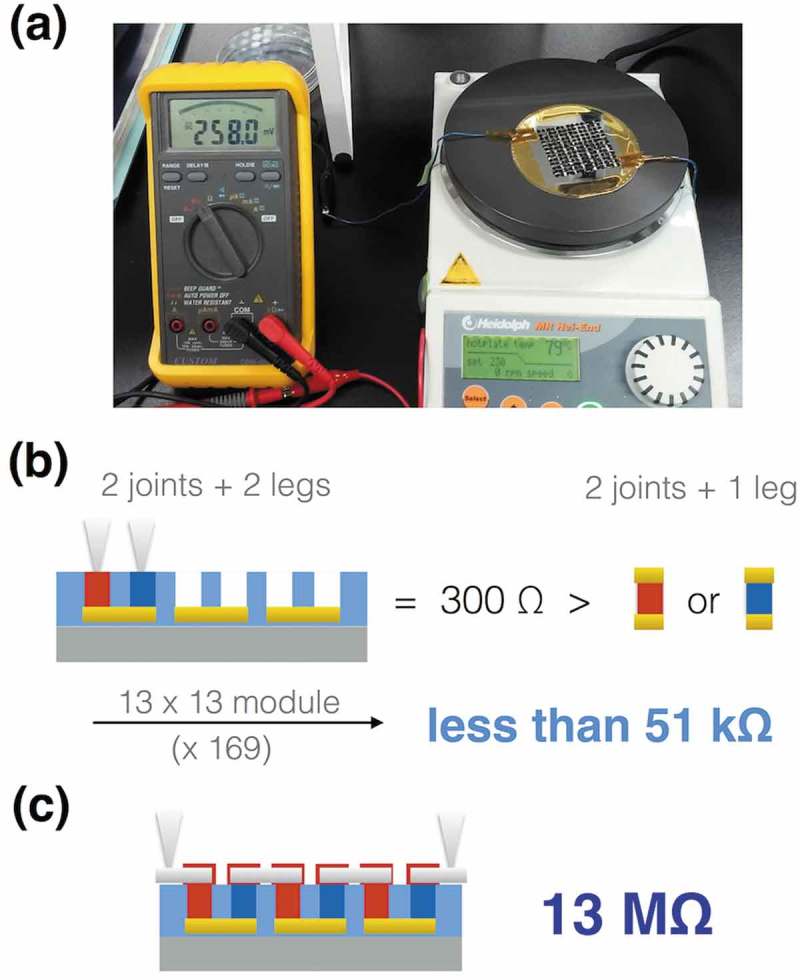
The organic π-type TE modules finalized with ECA: (a) photograph of the module achieving 250 mV; (b) schematic image of the single π-unit measurement and the estimated total resistance based on the measurement assuming the same contact resistance at the upper electrodes and at the bottom electrodes; (c) schematic image of the module measurement and the observed total resistance.

To achieve 250 mV on the organic π-type TE module, we next examined as-received PEDOT:PSS as an ECA with Al foil electrodes, because PEDOT:PSS was the base material of the p-type TE legs, and because it was water soluble and hence did not penetrate into the hydrophobic n-type TE legs. After individually adhering 85 Al electrodes with as-received PEDOT:PSS, the output voltage finally reached 250 mV at 80 ºC (Figure 9(a)). Assuming the same contact resistances at the upper electrodes and at the bottom electrodes, we had roughly calculated to be less than 50 kΩ in the total resistance because the single π-unit gives 300 Ω without the upper electrodes (Figure 9(b)). Nevertheless, the resistance reached 13 MΩ in total (Figure 9(c)). The observed resistance does not change even when using Ag paste as the ECA, where we quickly dried the Ag paste to prevent penetration into the n-type TE legs. We suppose that the difference between the calculated and observed resistances is caused by the different situations during the contact formations. The bottom electrodes are covered with the solution-mixed TE materials, in which case, the TE materials would deposit on the bottom electrodes as the unit levels and then the solution vapors can escape to the outside above the TE material layers. By contrast, the upper electrodes are put on top of the solution-mixed ECAs to encapsulate the solutions. In this case, the solution vapors necessitate the creation of outlets. Thus, the created outlets may damage the electrical contacts between the TE materials and the upper electrodes. This suggests a difficulty to reduce the contact resistance.

**Figure 10.**
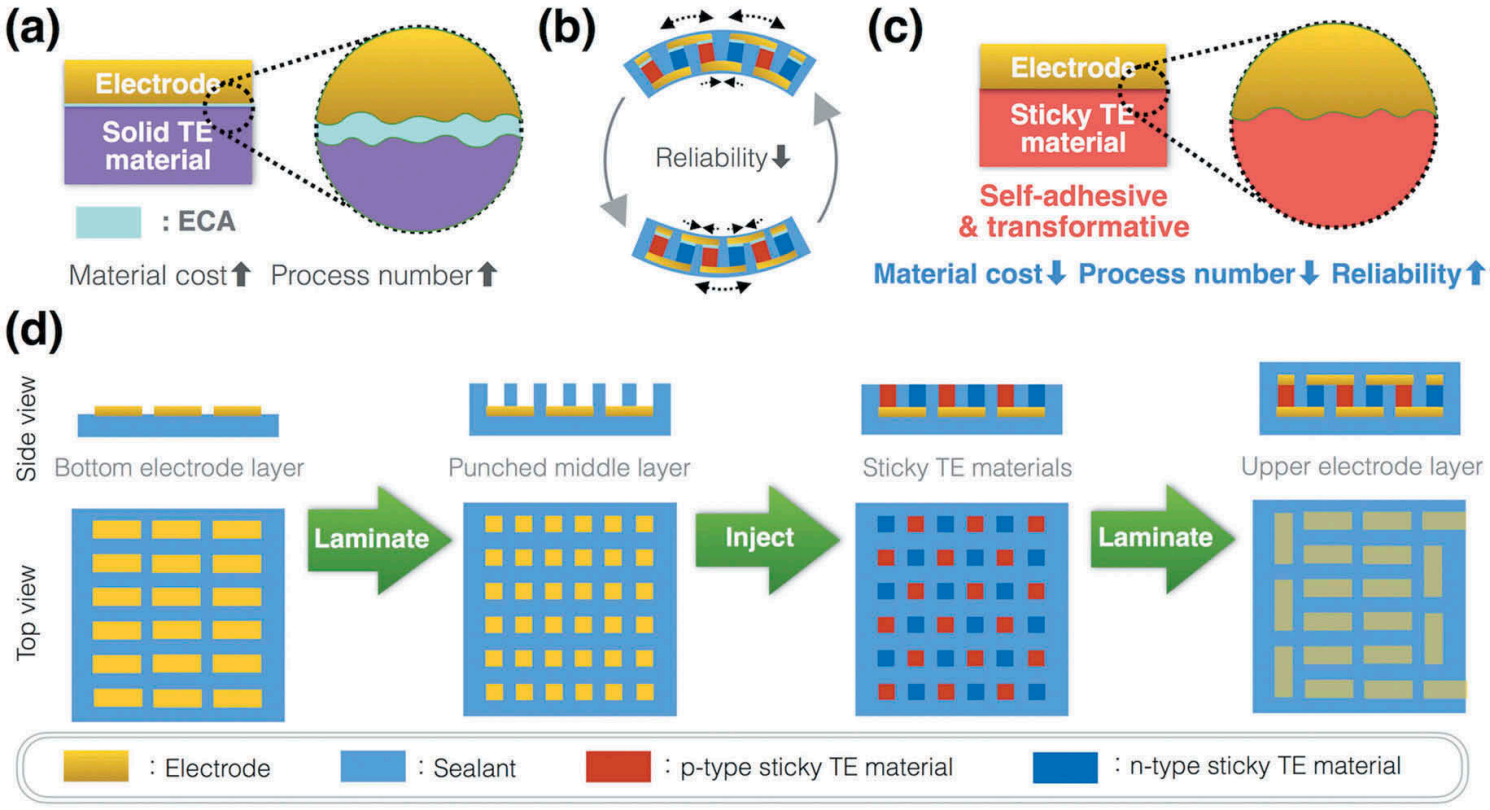
Expected benefits of sticky TE materials: (a) ECAs assist physical contact between solid TE materials and electrodes but form two measurable contact resistances due to low *σ* of ECAs; (b) Flexible π-type TE modules cause huge mechanical stress on the joints when bent because of the thickness to create Δ*T*; (c) Sticky TE materials adhere to the electrodes and absorb the mechanical stress; (d) The sticky TE materials enable a simple three-step fabrication process of reliable flexible TE sheets at reasonable material cost.

Additionally, the *σ* of ECAs strongly affects the total contact resistance because ECAs have two contacting interfaces with electrodes and with TE legs even though it assists the physical contact between electrodes and TE legs (Figure 10(a)). Contact resistance depends on *σ* of the two contacting materials; for example, semiconductor devices form high *σ* heavily doped regions under electrodes to make good electric contacts. When fabricating inorganic TE modules, we can braze electrodes and inorganic TE legs with filler metals to make good electric contact. Since organic materials cannot resist the brazing temperature, we need to use less conductive ECAs than brazed metals. Consequently, we cannot achieve good electric contacts in this scheme for organic π-type TE modules. To move the research of flexible TE modules forward, we also need to consider the other factors. In addition to the above disadvantage on contact resistance, ECAs do not have enough mechanical strength to hold the π-type TE module structure especially when the module is bent as a flexible module. Comparing the thickness of organic active layer ~100 nm in the other organic devices, such as organic light diodes and organic solar cells [20,21], we can expect that the TE leg thickness over 20 µm causes huge mechanical stress on the joints (Figure 10(b)). Furthermore, ECAs are generally expensive though this non-vacuum process should be economic. From a viewpoint to utilize the economic process, herein, we state a working hypothesis: low*-κ* TE materials like organics additionally acquire adhesive and transformable features; namely, sticky TE materials, in order that we can overcome the obstacles above on the flexible π-type TE modules. The low*-κ* feature is necessary for flexible sheet-type TE modules to maintain the *ΔT*. Sticky TE materials are favored as pressure-sensitive adhesives because they can adhere to electrodes after simple pressing [22]. By adhering to the electrodes and absorbing the mechanical stress, sticky TE materials enable a simple three-step fabrication process of reliable flexible TE sheets without expensive ECAs (Figure 10(c), (d)).

## 4. Conclusions

Due to the huge demand for energy harvesting from heat elements of various shapes, flexible TEs face a good opportunity to contribute to our society based on the technical advantage in energy conversion efficiency at low temperatures. In the traditional style of TE research, scientists have surveyed high-performance TE materials first, and then adjusted the TE module structures and the fabrication processes according to the material properties. Following this style, we have optimized the performances of organic TE materials first and then fabricated the organic TE modules. However, this research suggests that TE materials must be inversely designed from the viewpoints of the expected module structures and mass-production processes, especially for the purpose of energy harvesting. Taking a position to mass-produce flexible TE sheets at reasonable cost, herein; we propose a working hypothesis of sticky TE materials.
