# Supplementary material for: Organic π-type thermoelectric module supported by photolithographic mold: a working hypothesis of sticky thermoelectric materials
Source: Sci Technol Adv Mater. 2018 Jul 17;19(1):517–25. doi: 10.1080/14686996.2018.1487239 (PMC6052422; doi:10.1080/14686996.2018.1487239)
Supplement: Supplemental Material [file TSTA_A_1487239_SM4336.pdf]

Supplemental material for

Organic π-type thermoelectric module supported by photolithographic mold: a working hypothesis of sticky thermoelectric materials

Norifusa Satoh[a,*], Masaji Otsuka[a], Tomoko Ohki[a], Akihiko Ohi[a], Yasuaki Sakurai[b], Yukihiko Yamashita[b], Takao Mori[a,*]

[a]*International Center for Materials Nanoarchitectonics, National Institute for Materials Science (NIMS), Tsukuba, Japan*

[b]*Denka Innovation Center, Denka Company Limited, Tokyo, Japan*

*e-mail: SATOH.Norifusa@nims.go.jp, Mori.Takao@nims.go.jp

Table S1. Output voltage of the single π-unit of as-received PEDOT:PSS and the ball-milled TTF-TCNQ mixed with PVC at different ratios.

PVC/TTF-TCNQ	80 ºC	90 ºC	100 ºC
1/3	0.5 mV	0.5 mV	0.6 mV
1/12	0.9 mV	0.8 mV	1.0 mV
0	0.8 mV	1.1 mV	1.4 mV

Table S2. TE performances of PEDOT:PSS dedoped by KW-1000S.

	pH 1 (as-received)	pH 4	pH 7	pH 8
S (μV/K)	14.9	19.6	20.1	21.0
σ (kS/m)	1.24	0.231	0.513	0.762
PF (μW/mK^2)	0.275	0.0888	0.207	0.336

Table S3. TE performances of the dedoped PEDOT:PSS after the addition of different volume of DMSO per 1 ml PEDOT:PSS solution.

	0 μl/ml	10 μl/ml	20 μl/ml	30 μl/ml
S (μV/K)	21.0	21.6	21.6	21.2
σ (kS/m)	0.762	7.40	4.99	2.89
PF (μW/mK^2)	0.336	3.45	2.33	1.30

Table S4. Output voltage of the single π-unit of the ball-milled TTF-TCNQ and as-received PEDOT:PSS or the dedoped PEDOT:PSS after the addition of different volume of DMSO per 1 ml PEDOT:PSS solution.

	80 ºC	90 ºC	100 ºC	110 ºC	120 ºC	130 ºC
as-received	0.8 mV	1.1 mV	1.4 mV	1.1 mV	1.5 mV	1.6 mV
10 μl/ml	1.3 mV	1.5 mV	1.9 mV	2.2 mV	2.7 mV	3.1 mV
20 μl/ml	1.4 mV	1.7 mV	2.1 mV	2.3 mV	2.8 mV	2.9 mV
30 μl/ml	1.4 mV	1.7 mV	1.9 mV	2.2 mV	2.7 mV	2.8 mV
